# Correlation Between Residual Displacement and Osteonecrosis of the Femoral Head Following Cannulated Screw Fixation of Femoral Neck Fractures

**DOI:** 10.1097/MD.0000000000002139

**Published:** 2015-10-30

**Authors:** Chen Wang, Gui-Jun Xu, Zhe Han, Xuan Jiang, Cheng-Bao Zhang, Qiang Dong, Jian-Xiong Ma, Xin-Long Ma

**Affiliations:** From the Department of Orthopedics, Tianjin Hospital (CW, G-JX, ZH, X-LM); Tianjin Medical University (XJ, C-BZ); Department of Traumatic Orthopedics (QD); and Biomechanics Labs of Orthopedics Institute, Tianjin Hospital, Tianjin, People's Republic of China (J-XM).

## Abstract

The aim of the study was to introduce a new method for measuring the residual displacement of the femoral head after internal fixation and explore the relationship between residual displacement and osteonecrosis with femoral head, and to evaluate the risk factors associated with osteonecrosis of the femoral head in patients with femoral neck fractures treated by closed reduction and percutaneous cannulated screw fixation.

One hundred and fifty patients who sustained intracapsular femoral neck fractures between January 2011 and April 2013 were enrolled in the study. All were treated with closed reduction and percutaneous cannulated screw internal fixation. The residual displacement of the femoral head after surgery was measured by 3-dimensional reconstruction that evaluated the quality of the reduction. Other data that might affect prognosis were also obtained from outpatient follow-up, telephone calls, or case reviews. Multivariate logistic regression analysis was applied to assess the intrinsic relationship between the risk factors and the osteonecrosis of the femoral head.

Osteonecrosis of the femoral head occurred in 27 patients (18%). Significant differences were observed regarding the residual displacement of the femoral head and the preoperative Garden classification. Moreover, we found more or less residual displacement of femoral head in all patients with high quality of reduction based on x-ray by the new technique. There was a close relationship between residual displacement and ONFH.

There exists limitation to evaluate the quality of reduction by x-ray. Three-dimensional reconstruction and digital measurement, as a new method, is a more accurate method to assess the quality of reduction. Residual displacement of the femoral head and the preoperative Garden classification were risk factors for osteonecrosis of the femoral head. High-quality reduction was necessary to avoid complications.

## INTRODUCTION

Femoral neck fracture is a severe traumatic disease approximately comprising >50% of hip fractures, which mainly occur in the elderly after falls.^1^ In the USA, the incidence of femoral neck fracture was 63.3/million in women and 27.7/million in men.^2^ Although the detailed epidemiologic data are unknown in China, it has been estimated that a substantial number of individuals sustain femoral neck fractures. With aging of the population and the modernization of society, the incidence of femoral neck fracture tends to rise. Closed reduction and percutaneous cannulated screw internal fixation is the common surgical treatment procedure especially for young patients. Osteonecrosis of the femoral head (ONFH) and nonunion are the mayor complications following femoral neck fractures, which cause morbidity and economic burdens.^3^ With implant advancements and further understanding of femoral neck fractures, nonunion has improved significantly, which had occurred at a rate as high as 90%.^4^ However, ONFH still occurred frequently.^5^ It was reported that the frequency of osteonecrosis following cannulated screw internal fixation was ∼20%.^6^

In published studies, the quality of the reduction was considered to be significantly associated with ONFH.^7–9^ Generally, the judgment regarding the quality of the reduction was based on plain x-rays. Lowell noted that an ideal reduction was attained if an S-shaped curve was produced in the anteroposterior and lateral planes.^10^ Garden indicated that high-quality reduction was achieved when the alignment index was between 155° and 180° in both planes.^11^ The risk of ONFH would increase if a poor reduction were obtained.

Recently, it was reported that ONFH still occurred in some patients in whom the quality of the reduction was considered acceptable, according to the follow-up data. Therefore, we questioned whether it was appropriate to assess the reduction quality based on the 2-dimensional x-rays. Currently, no superior method existed to measure the residual displacement of the femoral head. A new method that was published in 2013^12^ was developed by our team to measure the preoperative displacement of Garden stage I fractures based on 3-dimensional reconstruction software. In our study, Garden stage I femoral neck fractures were found with some degrees of displacement that was invisible in plain x-ray. This method was applied to assess the residual displacement in the present study. Unlike the Garden alignment index proposed in 1961,^11^ the current assessment was based on the 3-dimensional model that provided measurements in a 3-dimensional plane. Therefore, we are able to describe the residual displacement of the femoral head precisely. Moreover, the new technique inspires us to reunderstand residual displacement of femoral head and re-evaluate postoperative or even intraoperative reduction of femoral neck fracture.

The purpose of the present study is to introduce a new method to measure the residual displacement of the femoral head, which was applied to assess the reduction quality and to evaluate the risk factors associated with ONFH in patients with femoral neck fractures who were treated with closed reduction and compression cannulated screw internal fixation. Although single-factor analysis was reported in previous studies, some confounding factors might affect the results. Thus, a multiple-factor comprehensive analysis was performed to further explore the relationship between the risk factors and ONFH.

## METHODS

The study enrolled patients who sustained femoral neck fractures between January 2011 and April 2013 and treated by closed reduction and percutaneous cannulated screw internal fixation in the traumatology department at Tianjin Hospital. The treatment strategy for femoral neck fracture was based on the guideline raised by American Academy of Orthopaedic Surgeons.^13^ The study was approved by the ethics committee of Tianjin Hospital. The follow-up data included age, gender, length of stay, fracture laterality, mechanism of injury, procedure delay, duration of surgery, implant configuration, interval to full weight-bearing, preoperative Garden classification, preoperative traction, postoperative visual analog scale (VAS), Parker score,^14^ implant status, residual displacement, and the occurrence of ONFH. All the data were collected through outpatient follow-up, telephone calls, or case reviews.

The patients who participated in the process should have met the following inclusion criteria: (1) age >20 years, (2) diagnosis of intracapsular femoral neck fracture, (3) treatment by closed reduction and percutaneous compression cannulated screw internal fixation, and (4) duration of follow-up >1.5 years. The exclusion criteria were as follows: (1) incomplete case data or image data, (2) old fractures or pathologic fractures, (3) bilateral fractures, (4) nonunion, and (5) postoperative hormone use. Finally, 150 patients were enrolled in this study.

**FIGURE 1:**
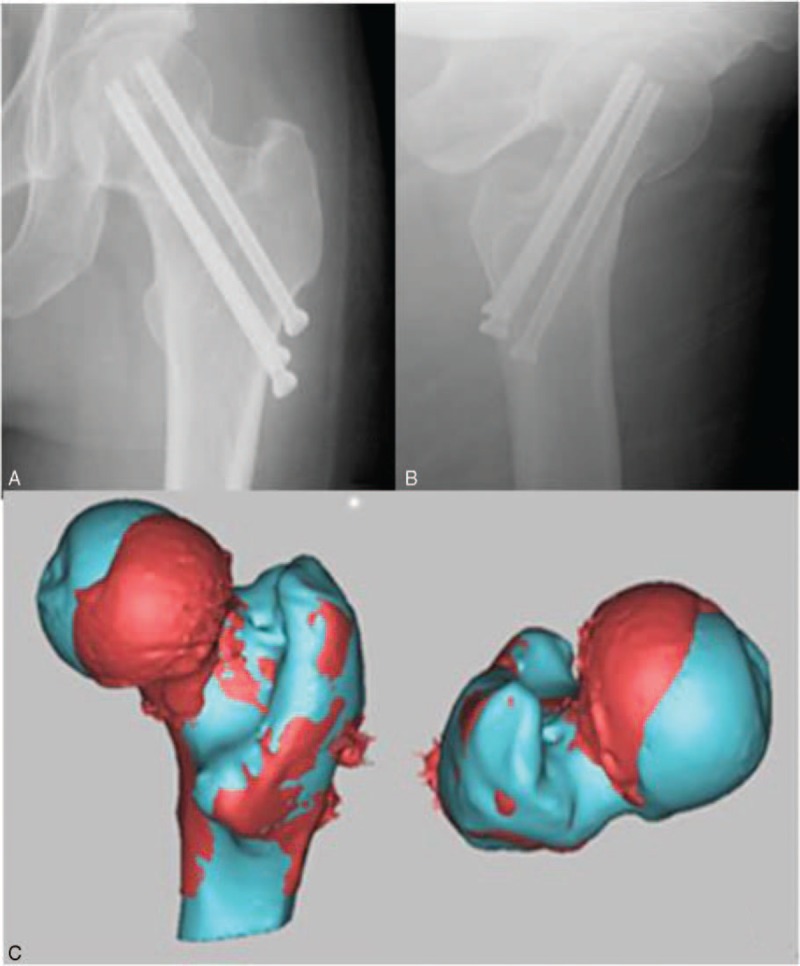
Antero-posterior and lateral postoperative radiograph of a left femoral neck fracture in a 55-year-old man (A and B), front and top views of proximal femoral 3-dimensional model and mirror model (C).

**FIGURE 2:**
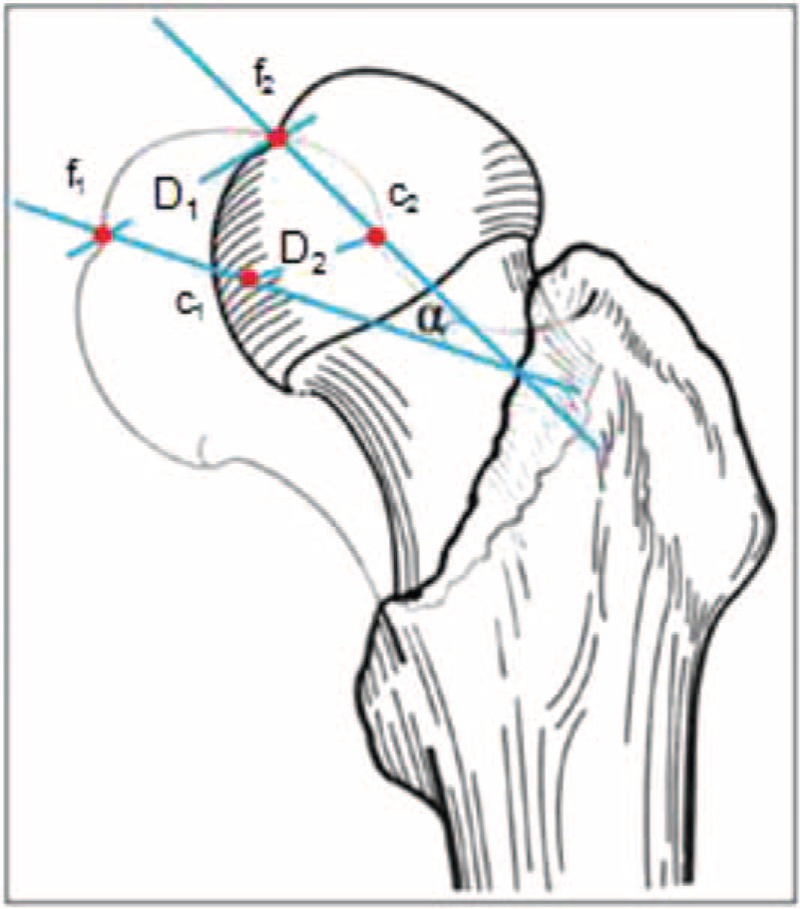
Measuring method for spatial displacements in the proximal femur.

The original CT data were produced by a Siemens Somatom Sensation 16 CT scanner. The scan area was the zone between 30 mm superior to the femoral head and 50 mm inferior to the midpoint of the lesser trochanter. Then, the CT data (DICOM) were imported to the 3-dimensional software, which generated 3-dimensional (3D) models of the bilateral proximal femurs. A mirror model of the fracture side was produced by the mirror function of the software. Then, the normal femur was superimposed on the mirror model (Fig. 1) and a new mask was formed in cross-sectional images. Two marking points were identified for measuring the residual femoral head displacement, which were the center of the femoral head and the deepest point in the fovea of the femoral head. The marking points were selected after the femoral head was sphere fit by the software. The following parameters were calculated to evaluate the residual displacement of the femoral head after the surgical procedure: D_1_: the distance between the points in the center of the femoral head in the 3D model (point c_1_) and in its mirror model (point c_2_), and D_2_: the distance between the points in the deepest femoral head fovea in the 3D model (point f_1_) and in its mirror model (point f_2_). Two lines were drawn through point c_1_and point f_1_ and through point c_2_ and point f_2_. An angle (α) was formed by the lines and was recorded to describe the rotation of the femoral head postoperatively (Fig. 2).

IBM-SPSS Version 19 statistical software was applied to analyze the data. A chi-square test was used to assess the significant differences of single factors between the ONFH group and the non-ONFH group with *P* < 0.05 considered a significant difference. When factors were considered significant between groups, multivariate logistic regression analysis was applied to calculate the regression model. The occurrence of ONHF or its absence was defined as a dependent variable, whereas the risk factors were defined as independent variables.

## RESULTS

There were 467 patients sustained a femoral neck fracture between January 2011 and April 2013. A total of 284 patients received hip arthroplasty and 183 patients received closed reduction and percutaneous screw fixation. After inclusion exclusion criteria, 150 patients were enrolled in the trial. According to age, 24 patients were between 20 and 40 years, 94 were between 40 and 60 years, and 32 were between 60 and 80 years. The average duration of follow-up was 37.4 ± 5.2 months (from 34 months to 41 months) and by gender, 62 patients were males and 88 were females. According to the Garden classification, 23 patients were Garden stage I, 30 were Garden stage II, 42 were Garden stage III, and 55 were Garden stage IV. The surgical delay was <24 h in 27 cases and >24 h in 123 cases. The time to weight-bearing was <3 months in 53 cases and >3 months in 97 cases. The quality of the reduction was considered to be acceptable in all patients based on the postoperative x-rays as judged by a senior radiologist. Concerning medical diseases, 29 patients had hypertension, 17 patients had type II diabetes mellitus, and 12 patients had coronary heart disease.

### The Occurrence of ONFH

**FIGURE 3:**
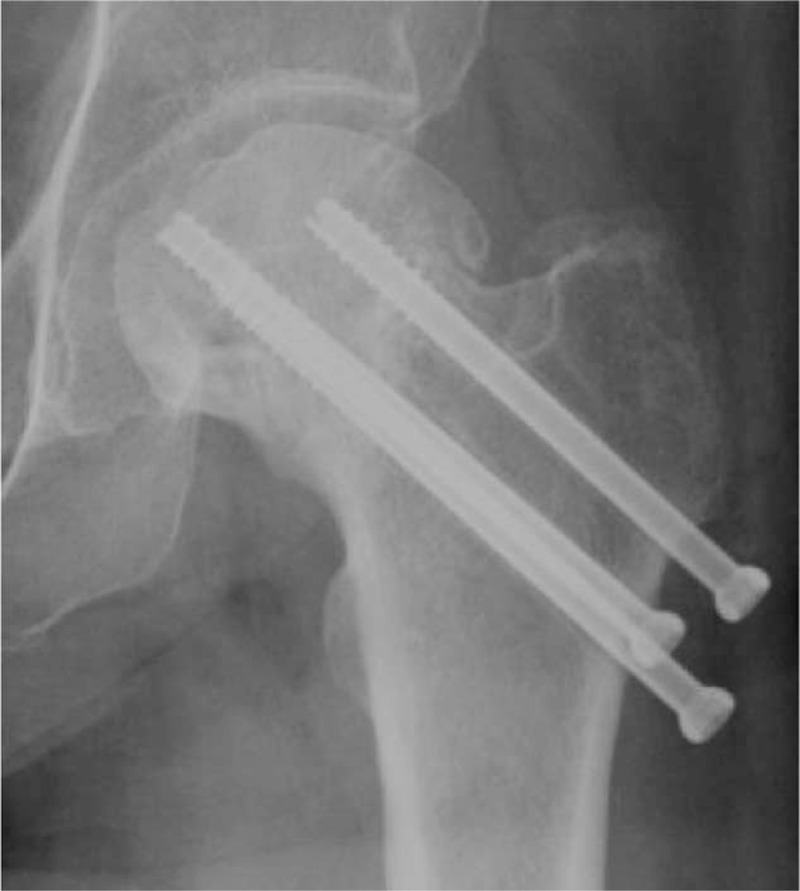
Antero-posterior radiograph at 3 years postoperatively showing the ONFH.ONFH = osteonecrosis of femoral head.

**FIGURE 4:**
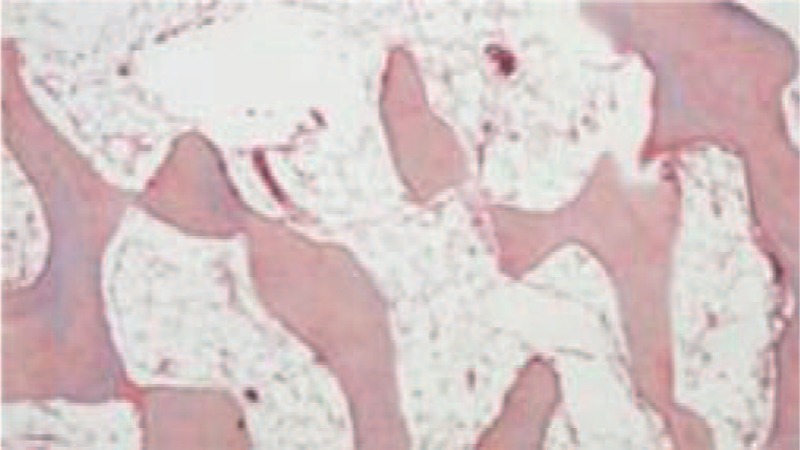
Pathological report indicated ONFH. ONFH = osteonecrosis of femoral head.

The diagnosis of ONFH was based on clinical symptoms and the imaging features by x-ray, CT, MRI, or postoperative pathological report (Fig. 3 and Fig. 4). Osteonecrosis of femoral head was diagnosed in 18% or 27 patients, 15 of whom subsequently received total hip arthroplasty. By gender, 10 were males and 17 were females. The average age was 51.9 ± 9.9 years. The average duration of the ONFH was 1.4 ± 0.7 years after surgery. Only 1 patient was Garden stage I, whereas 4 were Garden stage II, 10 were Garden stage III, and 12 were Garden stage IV. The time to weight-bearing was <3 months in 9 cases and >3 months in 18 cases. The cannulated screws were removed within 2 years in 9 of ONFH group and 9 of them received preoperative traction. The Parker score was 7.12 ± 3.23 in the ONFH group 1 year after surgery and 8.52 ± 0.93 in the non-ONFH group with an overall mean of 8.28 ± 1.15.

### Measurement of Residual Displacement of the Femoral Head

The measurements of the displacement of the center of the femoral head, the displacement of the deepest of the femoral head foveae, and the rotational displacement were shown in Table 1. The average displacement of the center of the femoral head was 8.71 ± 2.89 mm in the ONFH group and 6.66 ± 3.04 mm in the non-ONFH group. The average displacement of the deepest of the femoral head foveae was 12.81 ± 4.73 mm in the ONFH group and 10.82 ± 4.33 mm in the non-ONFH group. The rotational displacement was 19.63 ± 7.08° in the ONFH group compared to 16.81 ± 6.52° in the non-ONFH group.

**TABLE 1:**
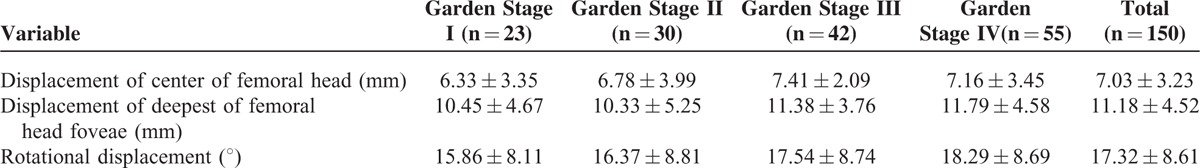
Measurement of Postoperative Displacement of Femoral Head

### Outcome for Single-Factor Analysis

A chi square test was used to test the significant differences between single factors and ONFH. The factors were age, gender, laterality of fractures, mechanism of injury, configuration of implants, preoperative Garden classification, preoperative traction, implant status, length of stay, procedure delay, duration of surgery, postoperative VAS, interval to full weight-bearing, and residual displacement of the femoral head. The results were shown in Table 2. Significant differences were found regarding the displacement of the center of the femoral head, the displacement of the deepest of the femoral head foveae, the rotational displacement, and the preoperative Garden classification between the ONFH group and the non-ONFH group.

**TABLE 2:**
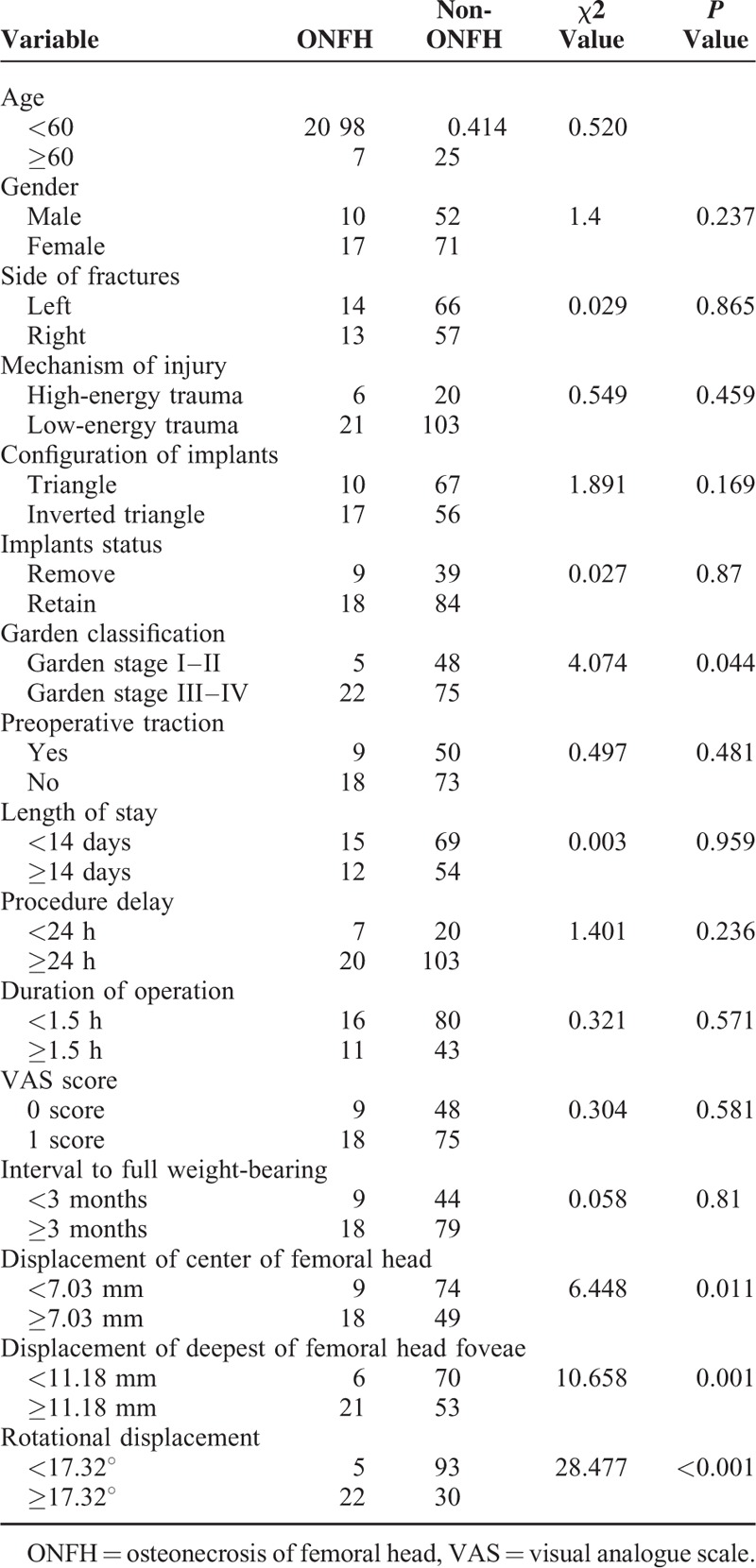
Chi Square Test of Single Factors

### Multiple logistic regression analysis

Factors that were considered significant differences associated with ONFH according to the chi square test were investigated by a multiple logistic regression analysis that was used to calculate a regression equation (Table 3 and Table 4). The analysis results indicated that the displacement of the center of the femoral head (OR = 2.989, *P* = 0.029), the displacement of the deepest of the femoral head foveae (OR = 3.352, *P* = 0.044), the rotational displacement (OR = 3.557, *P* = 0.020), and the preoperative Garden classification (OR = 3.740, *P* = 0.029) were significantly correlated with the occurrence of ONFH.

**TABLE 3:**
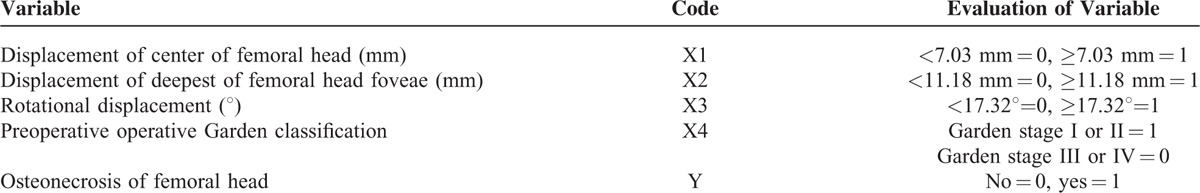
List of Variables for Evaluation

**TABLE 4:**
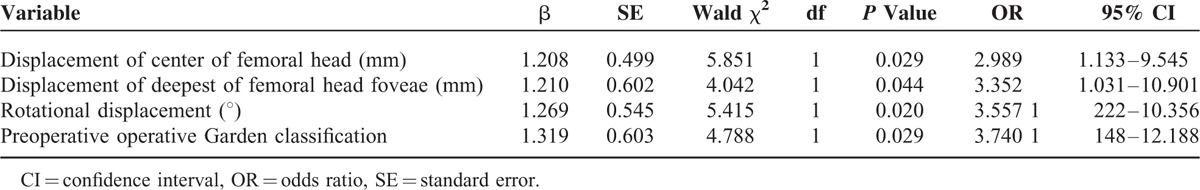
Result of Multivariate Logistic Regression Analysis

## DISCUSSION

The most important findings of the present study were that the occurrence of ONFH following internal fixation was significantly associated with residual displacement of the femoral head and the preoperative Garden classification, which are in accordance with the published literature. No significant differences were found between the groups regarding age, gender, fracture laterality, mechanism of injury, implant configuration, preoperative Garden classification, preoperative traction, implant status, length of stay, procedure delay, duration of surgery, postoperative VAS, and interval to full weight-bearing. Additionally, it was efficient to assess the reduction quality by 3-dimensional reconstruction.

The clinical application of compression cannulated screws for femoral neck fractures has been a breakthrough that has decreased the risk of nonunion significantly. However, ONFH still occurred frequently, that is, in nearly 20% of cases in published studies, which was slightly higher than our rate (18%). Perhaps the slight difference observed in our group occurred because some patients with severe displacement of the femoral head (Garden stages III and IV) received total hip arthroplasty directly.

In 1961, Garden classified femoral neck fractures into nondisplaced fractures (Garden stages I and II) and displaced fractures (Garden stages III and IV) based on plain x-rays and this classification was widely used throughout the world, which guided the fracture treatment and assessed the prognosis. We found that the rate of ONFH in patients with Garden stages I and II (9.4%) was lower than that of Garden stages III and IV (18.6%), which was in accordance with published studies.^7,8^ The reason for the higher incidence of ONFH was that the blood supply of the femoral head was more likely to be damaged with more severe degrees of preoperative displacement. Moreover, regarding the fractures that had been diagnosed as nondisplaced according to the Garden classification, we confirmed that they were not nondisplaced after surgery according to the 3-dimensional reconstruction. Thus, we hypothesized that Garden stage I and II fractures were displaced fractures. In previous studies, we had concluded that Garden type I fractures were not nondisplaced, stable fractures. Garden Type II fractures should also be reconsidered in subsequent research. Because the Garden classification system was proposed based on 2-dimensional images, it could not precisely describe the preoperative displacement of the femoral head.

The medial and lateral femoral circumflex arteries provide at least 90% of the blood supply to the femoral head. These arteries would be damaged following femoral neck fracture, which could induce osteonecrosis.^15^ It was widely believed that the blood supply would be reconstructed effectively by anatomic reduction. Therefore, investigators have noted that it was crucial to perform anatomic reductions. When necessary, open reduction could be performed.^16^ Wang^17^ conducted a meta-analysis, pooling the results to evaluate the correlation between ONFH and various reductions, open and closed, for femoral neck fractures. The results indicated there was a significant difference in terms of ONFH between the 2 reduction types.

Generally, the assessment of the reduction quality was based on plain x-rays. Although convenient and economic, this assessment was based on 2-dimensional planes that could not precisely describe the displacement of the femoral head. Uncertainty in the distance and the angle between the x-ray tube and the projection center might also affect the observations. Furthermore, it was difficult to obtain an AP radiograph due to pain-related postural changes.^18^ Based on the present study, we found that residual displacement was more or less present in all cases. None had obtained an anatomic reduction. Our group questioned whether there were some limitations in assessing the quality of the reduction by this method. Three-dimensional reconstruction based on the CT workstation was also insufficient to depict the residual displacement of the femoral head because we were unable to isolate the femoral head from the hip joint. A superior method was introduced in the present study, which made it possible to establish a vivid 3-dimensional model based on the original CT data and to obtain measurements using digital methods.

Logistic regression analysis showed significant differences regarding the residual displacement between the groups. The incidence of ONFH would increase in situations where the residual displacement was above the overall average. Therefore, we recommended that the postoperative residual displacement should be below the average.

Presently, debate endures regarding the optimal time of internal fixation. It was widely believed that the blood supply could be reconstructed efficiently by early surgical intervention, which reduced postoperative complications. Jain^19^ reported that there was a significant increase in the occurrence of ONFH with surgery proceeding after 12 h from the injury. Conversely, Razik^20^ concluded that there was no significant difference between the time delay to internal fixation and ONFH. No correlation was found between the procedural delay and the ONFH rate in our study. However, early fixation remains recommended to promote fracture healing and early functional rehabilitation.

In this study, the use of preoperative traction remained controversial. The purpose of traction was to relieve pain, to provide immobilization, and to decrease the risk of redisplacement for displaced femoral neck fractures. Xiao^21^ reported that continuous traction would increase the intracapsular pressure, which decreased blood perfusion to the femoral head and caused ONFH. However, no significant difference was observed regarding preoperative traction between the ONFH group and the non-ONFH group in our study.

We found that ONFH occurred following removal of the cannulated screws in some cases. Other studies also reported a similar phenomenon,^22,23^ although the mechanism was unclear and various hypotheses were proposed. The stress was concentrated on the implants before their removal. At that time, the remodeling of the bone trabeculae had not been accomplished, and microfractures could have occurred due to stress concentration on the femoral neck and head after the removal of the implants. Concurrently, the artery that supplied the femoral neck and head could be damaged, which might have led to ONFH. Although no significant difference was found regarding the removal of implants between the groups, we recommended that cannulated screws should not be removed, except in symptomatic patients.

There were some limitations to our study that should be noted as follows: (1) the sample size was relatively small, which decreased the level of evidence; (2) for some older patients, total hip arthroplasty may be performed directly and they were excluded from the follow-up, which would affect the results; (3) various brands of implants and the preoperative bone quality were not taken into consideration, which might influence the ONFH outcome; and (4) the duration of follow-up was relatively brief. Despite these limitations, this was the first report to assess the quality of the reduction for femoral neck fractures by 3-dimensional reconstruction and then to analyze the risk factors correlated to ONFH by follow-up. Larger study population sizes and long-term follow-up are needed to further explore the risk factors and their correlation to ONFH in the future.
